# Gene expression in periodontal tissues following treatment

**DOI:** 10.1186/1755-8794-1-30

**Published:** 2008-07-07

**Authors:** Thomas Beikler, Ulrike Peters, Karola Prior, Martin Eisenacher, Thomas F Flemmig

**Affiliations:** 1 Department of Periodontics, University of Washington, Seattle, USA; 2 University of Veterinary Medicine, University of Hannover, Hannover, Germany; 3 Department of Periodontology, Westfalian-Wilhelms-University, Muenster, Germany; 4 Institute of Medical Proteomics, Ruhr-University, Bochum, Germany

## Abstract

**Background:**

In periodontitis, treatment aimed at controlling the periodontal biofilm infection results in a resolution of the clinical and histological signs of inflammation. Although the cell types found in periodontal tissues following treatment have been well described, information on gene expression is limited to few candidate genes. Therefore, the aim of the study was to determine the expression profiles of immune and inflammatory genes in periodontal tissues from sites with severe chronic periodontitis following periodontal therapy in order to identify genes involved in tissue homeostasis.

Gingival biopsies from 12 patients with severe chronic periodontitis were taken six to eight weeks following non-surgical periodontal therapy, and from 11 healthy controls. As internal standard, RNA of an immortalized human keratinocyte line (HaCaT) was used. Total RNA was subjected to gene expression profiling using a commercially available microarray system focusing on inflammation-related genes. Post-hoc confirmation of selected genes was done by Realtime-PCR.

**Results:**

Out of the 136 genes analyzed, the 5% most strongly expressed genes compared to healthy controls were Interleukin-12A (IL-12A), Versican (CSPG-2), Matrixmetalloproteinase-1 (MMP-1), Down syndrome critical region protein-1 (DSCR-1), Macrophage inflammatory protein-2β (Cxcl-3), Inhibitor of apoptosis protein-1 (BIRC-1), Cluster of differentiation antigen 38 (CD38), Regulator of G-protein signalling-1 (RGS-1), and Finkel-Biskis-Jinkins murine osteosarcoma virus oncogene (C-FOS); the 5% least strongly expressed genes were Receptor-interacting Serine/Threonine Kinase-2 (RIP-2), Complement component 3 (C3), Prostaglandin-endoperoxide synthase-2 (COX-2), Interleukin-8 (IL-8), Endothelin-1 (EDN-1), Plasminogen activator inhibitor type-2 (PAI-2), Matrix-metalloproteinase-14 (MMP-14), and Interferon regulating factor-7 (IRF-7).

**Conclusion:**

Gene expression profiles found in periodontal tissues following therapy indicate activation of pathways that regulate tissue damage and repair.

## Background

Non-surgical periodontal therapy has been shown to result in a significant reduction of pocket probing depths and bleeding on probing, and attachment gain [1-5]. When combined with periodontal maintenance a long-term stability of periodontal conditions, i.e. a significantly reduced incidence of additional attachment loss and reduction in bleeding on probing (BOP), is achievable and has been shown even in residual pockets with probing depths of greater than 7 mm [4,6]. These clinical data indicate that significant changes in the immune/inflammatory response occur following treatment. However, it is unknown, whether the clinical signs following periodontal therapy are associated with an expression profile of inflammatory and immunological genes that is compatible with periodontal health.

Despite the large amount of clinical data, changes in the inflammatory status following non-surgical therapy have only been sparsely studied in humans. Histological studies have indicated that the gingival infiltrate associated with dental plaque is mainly characterized by a B cells and plasma cells [7]. Following therapy, the density of CD19, CD20, CD30 and CD45RO positive cells were found to be reduced, while the overall relative number of CD3 positive cells remained unchanged [8-11]. Few studies that have attempted to analyze the expression of inflammation related genes following non-surgical therapy; they have been limited to selected genes, e.g. IL-1β[12,13], IFN-γ, IL-2, IL-4, IL-5, IL-6, and TNF-α [13]. A more comprehensive analysis of gene expression related to immune and inflammatory processes is, however, needed to fully appraise the genes involved in tissues homeostasis following therapy.

Therefore, the aim of this study was to analyze the expression profiles of a broad spectrum of genes associated to immune or inflammatory processes in the gingiva of periodontitis sites following periodontal therapy and to identify candidate genes that may serve as targets for new treatment or diagnostic strategies.

## Results

### Demographics and periodontal condition

Demographics and periodontal findings of enrolled periodontitis patients before and following treatment and of healthy controls are shown in Table 1. Overall, non-surgical periodontal therapy resulted in a reduction of bleeding on probing (BOP) from 58.6 ± 29.8 to 19.3 ± 13.4. The percentage of sites with PPD of 4 to 6 mm was reduced from 42.2 ± 13.8 to 30.6 ±18.9 and sites with initial PPD of 7 mm and greater from 18.9 ± 3.9 to 15.7 ± 5.8 following supra- and subgingival debridement.

**Table 1:** Demographics and periodontal conditions in the study subjects.

	**Test**	**Control**
Subjects (n)	12	11
Age (years)	46.6	38.5
± SD	11.7	5.6
Females	6	7
Smokers	4	3
Mean no. of teeth/subject ± SD	22.4 ± 5.9	27.36 ± 1.4
Mean % of sites with PPD 4–6 mm ± SD	42.2 ± 13.8	0
following SGD	30.6 ± 18.9	0
Mean % of sites with PPD ≥ 7 mm ± SD	18.9 ± 3.9	
following SGD	15.7 ± 5.8	
Mean % of sites with BOP ± SD	58.6 ± 29.8	
following SGD	19.3 ± 13.4	

SD = standard deviation, SGD = subgingival debridement.

### Gene expression profiles

**Figure 1:**
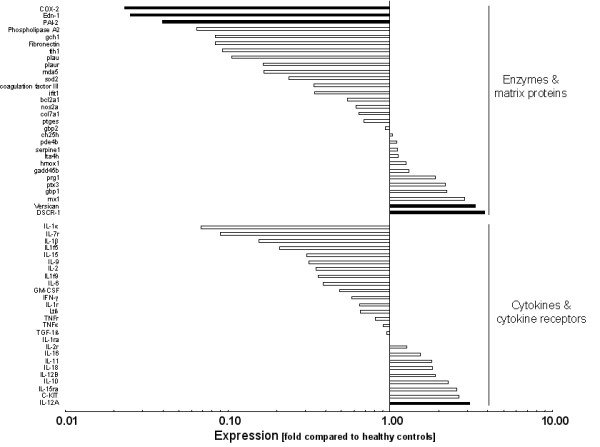
**Logarithmic depiction of gene groups and genes analyzed: Chemokines & chemokine receptors, complement system, acute phase proteins, adhesion molecules, Toll-like receptors, growth factors, and matrix-metalloproteinases.** Black bars represent the 5% strongest and the 5% weakest expressed genes. Data represent relative x-fold expression compared to healthy controls.

**Figure 2:**
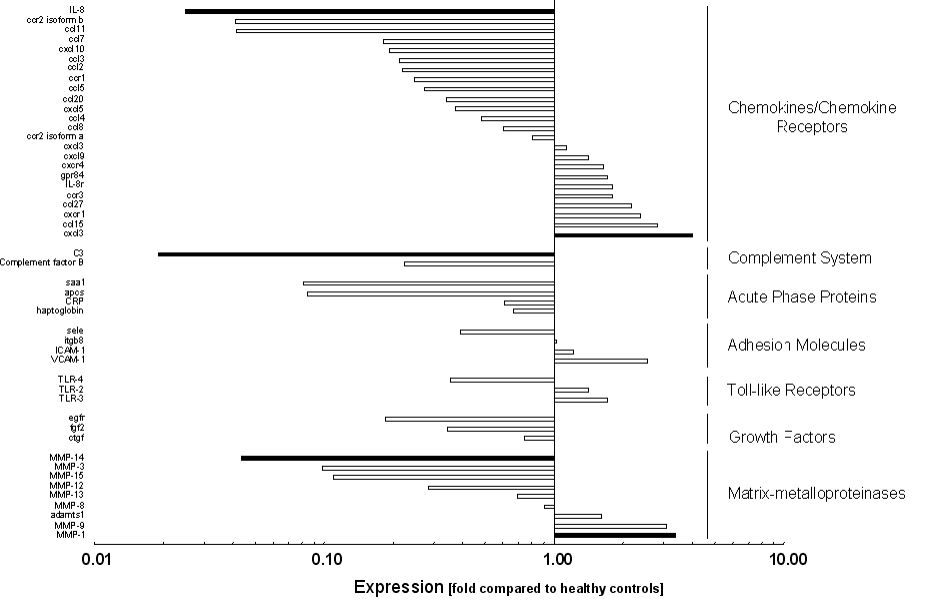
**Logarithmic depiction of gene groups and genes analyzed: Cytokines & cytokine receptors, enzymes & matrix proteins.** Black bars represent the 5% strongest and the 5% weakest expressed genes. Data represent relative x-fold expression compared to healthy controls.

**Figure 3:**
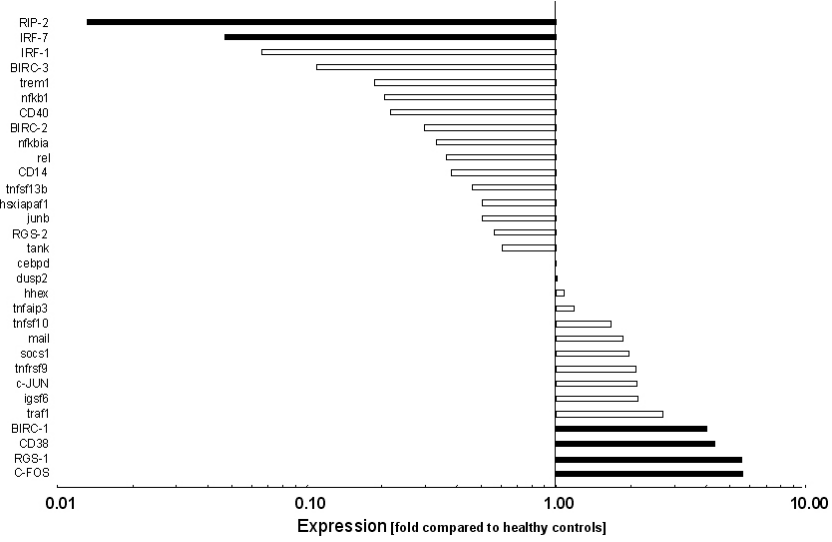
**Logarithmic depiction of gene groups and genes analyzed: Signal transduction & transcription factors.** Black bars represent the 5% strongest and the 5% weakest expressed genes. Data represent relative x-fold expression compared to healthy controls.

In gingival tissues from treated periodontitis sites, the 5% most strongly expressed genes were Interleukin-12A (IL-12A, NM_000882, 3.1-fold), Versican (CSPG-2, NM_004385, 3.35-fold), Matrix metalloproteinase-1 (MMP-1, NM_002421, 3.37-fold), Down syndrome critical region protein-1 (DSCR-1, NM_004414, 3.79-fold), Macrophage inflammatory protein-2beta (cxcl-3, NM_002090, 3.99-fold), Inhibitor of apoptosis protein-1 (BIRC-1, NM_004536, 4.04-fold), Cluster of differentiation antigen 38 (CD38, NM_001775, 4.43-fold), Regulator of G-protein signalling-1 (RGS-1, NM_002922, 5.53-fold), and Finkel-Biskis-Jinkins murine osteosarcoma virus oncogene (C-FOS, NM_005252, 5.65-fold). The 5% least expressed genes were Receptor-interacting serine/threonine kinase-2 (RIP-2, NM_003821, 0.01-fold), Complement component 3 (C3, NM_000064, 0.02-fold), Prostaglandin-endoperoxide synthase-2 (COX-2, NM_000963, 0.02-fold), Interleukin-8 (IL-8, NM_000584, 0.02-fold), Endothelin-1 (EDN-1, NM_001955, 0.03-fold), Plasminogen activator inhibitor type-2 (PAI-2, NM_000602, 0.04-fold), Matrixmetalloproteinase-14 (MMP-14, NM_004995, 0.04-fold), and Interferon regulating factor-7 (IRF-7, NM_00157, 0.05-fold) (Figure 1, 2, 3). The expression of the described genes was significantly different (p < 0.05) between treated periodontitis sites and healthy controls. The results of the Realtime-PCR are being shown in Table 2. Compared to the housekeeping genes, the 5 analyzed genes were up-regulated by a factor of 2.40 to 9.63.

**Table 2:** Results of the Realtime-PCR analysis.

	compared to NADH-dehydrogenase	compared to pyruvate-dehydrogenase
RGS-1	9.03	9.63
CD38	5.45	5.81
C-FOS	5.13	5.47
DSCR-1	3.23	3.44
CSPG-2	2.40	2.57

RGS-1 = Regulator of G-protein signalling-1; CD38 = Cluster of differentiation 38; C-FOS = Finkel-Biskis-Jinkins murine osteosarcoma virus oncogene; DSCR-1 = Down critical region protein 1; CSPG-2 = Versican.

## Discussion

An important finding of the study is that in periodontitis sites following non-surgical therapy the overall expression profile was downregulated compared to healthy controls. The diminished expression of inflammatory and immune related genes may indicate that periodontal therapy results in a strong reduction of the bacterial challenge thus resulting in a decreased stimulation of inflammatory/immune responses. In this regard the decreased expression of pro-inflammatory genes like IL-1 and IL-6 that have been demonstrated to be over-expressed in untreated periodontitis sites [14-16] may indicate a strong down-regulation of the pro-inflammatory immune response induced by periodontal treatment.

In most published microarray studies cutoffs of two to threefold up- or down-regulation have been used to define differential expression from controls [17]. For interpretation of the results, we even expanded this cutoff by focussing on the 5 percent most strongly up- or down-regulated genes to determine candidate genes that are associated to the regulation of tissue homeostasis following periodontal therapy.

Among the up-regulated genes, DSCR-1 inhibits the dephosphorylation of the nuclear factor of activated T-cells (NFAT) by calcineurin, which leads to decreased T-cell activation and cytokine production [18]. Moreover, DSCR-1 down-regulates transcription of several pro-inflammatory factors, including COX-2 and IL-8 [19,20], which were also down-regulated following periodontal therapy.

IL-12 is known to be mainly a product of activated inflammatory cells (monocytes, macrophages, neutrophils, microglia and dendritic cells) and induces the differentiation of T helper 1 (T_H_1) cells. The biologically active IL-12 (IL-12p70) is a heterodimeric cytokine that consists of two heterologous chains, named p40 and p35. The up-regulated expression of IL-12p35 found in the present study may indicate an enhanced production of IL-12 and thus may indicate a T_H_1 response in the treated periodontal tissues. An elevated IL-12 production has already been described in patients following periodontal therapy [21,22]. Also, the increased expression of CD38, which is found on early T-cell precursors and mature T cells upon mitogenic activation indicates a T-cell response [23].

Regulatory T-cells express high levels of Regulator of RGS-1 upon cell activation [24]. These regulatory T-cells counteract the collateral tissue damage initiated by an antimicrobial immune response [25]. The increased expression of RGS-1 following therapy may be a mechanism to limit the damage upon antigen-induced inflammation. The increased expression of BIRC-1, a potent inhibitor of activated caspase-3 and -7 [26] and the decreased expression of RIP-2, a strong activator of NF-κB and Fas receptor induced apoptosis [27] further support this notion. It has been described that in untreated periodontitis sites, the activity of caspase-3, -7, and Fas is increased compared to healthy tissues [28] and thus the up-regulation of BIRC-1 as well as decreased expression of RIP-2 may represent different protective host mechanisms to control apoptotic processes following periodontal therapy.

C-Fos, a member of the Fos family of transcription factors, together with Jun family members forms the group of AP-1 proteins, which, after dimerisation, bind to promoter and enhancer regions of target genes associated to cell proliferation, differentiation, and apoptosis [29]. Cellular processes that are characteristic to wound healing processes may also be seen following periodontal therapy.

CXCL-3 expression has been described to drastically increase after inoculation of *Porphyromonas gingivalis *in rats immunized against *P. gingivalis *[30]. IRF-7 is essential for the induction of a systemic type-I Interferon (IFN-alpha/beta) response for innate antiviral immunity as well as the induction of a CD8^+ ^T-cell response in adaptive immunity [31]. The decreased expression of IRF-7 may indicate a weak CD8^+ ^T-cell response and corroborates findings that have shown that CD8^+ ^cytotoxic T-cell-mediated immunity does not play a major role in periodontal tissue destruction [32].

The finding of an increased expression of MMP-1 corroborates previous findings in treated periodontal tissues [33]. CSPG-2 is a large chondroitin sulfate proteoglycan that has been found in periodontal tissues [34] and is known to be involved in tissue formation by capturing a temporal space for succeeding tissues/cells in embryonic tissue, cell adhesion, cell proliferation and cell migration [35]. Therefore, its increased expression fits into the context of healing/restructuring processes that take place following non-surgical periodontal treatment.

MMP-14 was strongly down-regulated, when compared with healthy controls. As this membrane bound proteinase has not been found to be over-expressed in gingival tissues from periodontitis sites [35], the role it plays for tissue homeostasis remains unclear. C3 is an acute phase reactant whose levels become elevated in response to acute and chronic inflammation or tissue injury [36]. The expression of C3 is up-regulated by pro-inflammatory cytokines such as IL-1, IL-6, IFN and TNF-α [37]. As these cytokines were expressed within normal range in periodontitis sites following periodontal therapy, the decreased expression of C3 may further indicate a normal inflammatory status that is reached following periodontal therapy. The same interpretation can be applied to the result of decreased expression of END-1 and PAI-2, since they are usually up-regulated when periodontal inflammation exists [38-40].

It should be pointed out that the results of this study are based on a relatively low number of specimens, which warrants cautious interpretation. Moreover, the cellular source of the observed expression patterns is unclear and could probably also reflect a different cellular composition of the tissues analyzed. Further in vivo and in vitro studies are needed to fully appraise cellular pathways and their interaction in periodontal tissues following therapy.

## Conclusion

Within the limits of the present study it may be concluded that in periodontitis sites following non-surgical periodontal therapy, the overall expression profile of immune and inflammatory genes was downregulated compared to healthy controls. Genes that were found to be up- or down-regulated compared to healthy controls are associated with the activation of tissue repair mechanisms and pathways that regulate tissue damage induced by the immune responses.

## Methods

### Study subjects

Twelve patients with periodontitis and eleven healthy controls were enrolled in the study at the Department of Periodontology, University of Muenster, Germany. Patients were included when they had untreated severe generalized chronic periodontitis [41] and equal to or more than 16 natural teeth. Patients with any of the following conditions were excluded from the study: requirement for prophylactic antibiotics; intake of any drugs; severe cardiovascular, hepatic, immune, renal, hematological, mental, inflammatory disorders other than periodontal disease or any other organ disorders. Subjects in the control group were systemically healthy and showed no signs of periodontitis as determined by the absence of probing depths greater than 3 mm and overt signs of gingival inflammation. All enrolled subjects gave written consent on a form approved by the Ethics Committee of the Medical Chamber of Westfalia-Lippe and the Medical Faculty of the Westfalian-Wilhelms-University, Muenster, Germany (approval number: 1Vbei, T. Beikler as PI).

### Periodontal therapy

**Figure 4:**
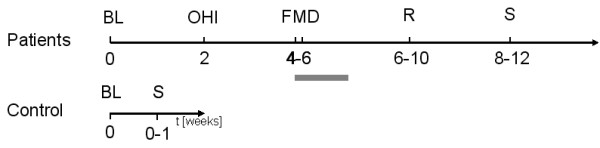
**Study design and timeline.** BL = Baseline, OHI = Oral hygiene instruction, FMD = Full mouth disinfection, R = Re-evaluation, S = Specimen. Grey bar represents mouth rinse with 0.12% Chlorhexidine (b.i.d. for 2 weeks).

All patients received oral hygiene instructions followed by full mouth supra- and subgingival debridement and adjunctive subgingival irrigation with 1.0% chlorhexidine digluconate gel. Full mouth debridement was performed in two appointments on two consecutive days and completeness of supra- and subgingival debridement, i.e., all pathologically exposed subgingival root surfaces feeling hard and smooth, was determined by using a fine explorer. Following therapy, patients were asked to rinse twice daily with 0.2% chlorhexidine digluconate solution for 14 days [42]. The complete study design is depicted in Figure 4.

### Gingival biopsies

**Figure 5:**
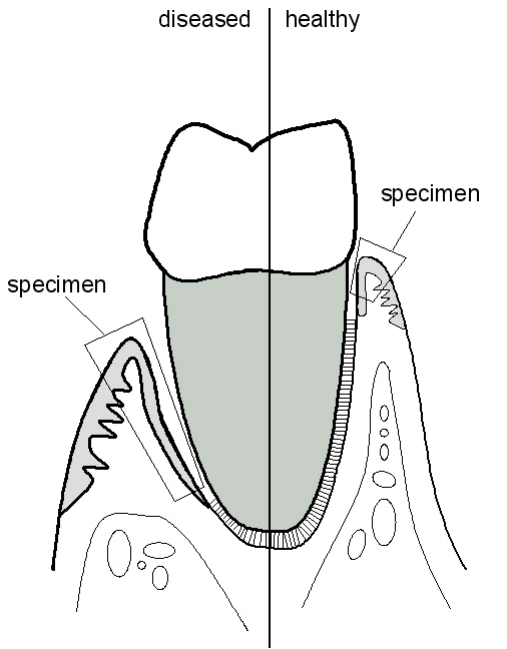
Schematic depiction of the specimen and tissue analyzed.

Six to eight weeks following full mouth debridement, gingival biopsies were obtained from periodontal sites of patients with residual pockets probing depth of 7 mm or greater. All of the patient samples and 7 out of the 11 healthy control specimens have been obtained by the first author. Four healthy control specimens have been obtained by another experienced periodontist (BE, see Acknowledgements) using the same standardized procedure in obtaining the biopsies and in processing the specimen. Under local anesthesia (Articain with 1:100.000 epinephrine), paracrestal incisions were made, and mucoperiosteal flaps were reflected. The excised gingival collar was then carefully removed from the roots and the alveolar process. Gingival biopsy comprised the oral gingival epithelium, pocket epithelium, junctional epithelium, and the interposed connective tissue (see Figure 5). In healthy controls, gingival biopsies were taken from the palatal area of premolars in a similar fashion, but without reflecting a mucogingival flap. Biopsy specimens were immediately placed into RNA-later (Qiagen Inc., Valencia, CA, USA) and stored at -20°C.

### Keratinocyte culture

An immortalized human keratinocyte line (HaCaT) was used as internal standard for RNA expression. Cells were cultured in M199 medium supplemented with 2 mM L-glutamine, 10% fetal calf serum, 10% horse serum and antibiotics, and grown at 37°C in a 5% CO2 atmosphere. They were allowed to grow to confluence and then harvested and subjected to RNA extraction as described below.

### RNA extraction, cDNA synthesis with labeling, and hybridization of microarray slides

Total RNA was isolated using RNeasy Protect Mini Kit (Qiagen Inc., Valencia, CA, USA) according to the manufacturer's instructions with the following modifications: Tissues were disrupted by a motor pestle (Novo-direct GmbH, Kehl, Germany) and homogenized with QIAshredder (Qiagen Inc., Valencia, CA, USA). The remaining DNA was removed using the RNase-Free DNase kit (Qiagen Inc., Valencia, CA, USA). Quality and concentration of RNA was analyzed with an Agilent 2100 Bioanalyzer (Agilent Technologies Inc., Palo Alto, CA, USA). Microarray analysis was performed using a human inflammation microarray (MWG Biotech, Martinsried, Germany), which was spotted with probes for 136 genes associated with inflammatory and immune response, 19 housekeeping genes, and five arabidopsis control oligonucleotides [43].

For hybridisation, 20 μg of total RNA originating from treated periodontitis sites and healthy gingival tissues and the same amount of RNA from HaCAT cells were reverse transcribed using the CyScribe First-Strand cDNA Labelling Kit (Amersham Inc., Piscataway, NJ, USA) according to the manufacturer's instructions. The cDNA of gingival tissues were labeled with Cy5 and the internal standard cDNA from HaCaT cells with Cy3. cDNA was cleaned using the GFX Purification kit (Amersham Inc., Piscataway, NJ, USA) according to the manufacturer's instructions. Microarrays (MWG Biotech, Martinsried, Germany) were hybridized at 42°C for 24 h under constant shaking. Probe array scanning and analysis was performed by MWG.

### Real-time-PCR

For the validation of the expression analysis real-time-PCR of five highly expressed genes (RGS-1, CD38, C-FOS, DSCR-1, CSPG-2) was performed. RNA was reverse transcribed into cDNA using SuperScript III first-strand synthesis System (Invitrogen GmbH, Karlsruhe, Germany) according to the manufacturer's instructions. Real-time-PCR was performed using the ABI PRISM 7700 Sequence detection system (Applied Biosystems, Foster City, California, USA) with TaqMan Primers and probes specific for the five above mentioned genes and two additional housekeeping genes (NADH-dehydrogenase and pyruvate-dehydrogenase). The reaction mixture contained 25 μl of TaqMan universal PCR master Mix, 2 μl of 10 μM of each primer, 2.5 μl of TaqMan probe and 2 μl of cDNA. It was adjusted to 22.5 μl with DNase/RNase free water. Conditions of the real-time-PCR were as follows: 2.0 min at 50°C (reverse transcription), 15 min at 95°C (RT inactivation and initial activation), and then 40 cycles of amplification for 15 sec at 95°C (denaturation), and 1 min at 60°C (annealing and extension). All heating and cooling steps were performed with a slope of 20°C/s. A single fluorescence reading at 530 nm was obtained for each sample at the extension step. Samples were analyzed in duplicates and averages were calculated for analysis of the expression ratios using REST-software [44].

### Statistics

Average expression values from treated periodontitis biopsies were divided by expression values from healthy gingival tissues. The 5% most strongly (3.1 to 5.65 fold) and the 5% least strongly (0.01 to 0.05 fold) expressed genes were identified, and Mann-Whitney ranksum tests was performed to determine differences between treated periodontitis sites and healthy controls.

## Authors' contributions

TB was PI on that project, wrote the grant and the protocol for the institutional review board, and provided most of the clinical samples. Moreover, he supervised all of the laboratory experiments and did the writing of the manuscript. UP and KP performed all of the laboratory experiments. ME did the statistical analysis. TFF was Co-PI on that project and was actively involved in data management, interpretation of the results, and writing of the manuscript. All authors have read and approved the final manuscript.

## Pre-publication history

The pre-publication history for this paper can be accessed here:


